# Chloroplast Genome Variation and Phylogenetic Relationships of *Lavandula angustifolia* and *Lavandula* × *intermedia*

**DOI:** 10.3390/ijms27219567

**Published:** 2026-10-26

**Authors:** A. V. Popov, K. O. Petrova, M. V. Patrushev, S. V. Toshchakov, F. S. Sharko

**Affiliations:** National Research Center “Kurchatov Institute”, Academician Kurchatov pl., 1, Moscow 123182, Russia

**Keywords:** *Lavandula angustifolia*, *Lavandula* × *intermedia*, assembly, annotation genome, de novo, chloroplast genome, phylogenetic analysis, taxonomic misidentification

## Abstract

Plant chloroplast genomes are characterized by high conservatism and a well-described organization, making them a convenient basis for analyzing evolutionary dynamics and constructing phylogenetic hypotheses. In this study, we performed de novo assembly and complete annotation of six chloroplast genomes from the genus *Lavandula*: four accessions of *L. angustifolia* and two hybrid samples of *Lavandula* × *intermedia*. The assembled genomes ranged from 151,431 to 151,480 bp, which is consistent with the expected chloroplast genome organization and indicates high structural stability within the genus. All six genomes exhibited the typical quadripartite structure of angiosperm chloroplast genomes, with a large single-copy (LSC) region of 82,682–82,837 bp, a small single-copy (SSC) region of 17,579–17,580 bp, and two inverted repeats (IRa and IRb) of 25,532–25,586 bp each. They contained 113 genes in total, including 79 protein-coding genes, 30 tRNA genes and 4 rRNA genes. Nucleotide diversity analysis revealed several variable intergenic regions that could serve as potential candidate molecular markers. Each sample harboured 56–57 simple sequence repeat (SSR) loci, unevenly distributed across the genome. Phylogenetic analysis based on whole chloroplast genome alignments confirmed the placement of the newly assembled *Lavandula* sequences within the family Lamiaceae. The six chloroplast genomes obtained in this work provide a robust reference set for future comparative genomic, phylogenetic and population-level studies in *Lavandula*.

## 1. Introduction

*Lavandula angustifolia* (lavender) is one of the most valuable aromatic and medicinal plants, with a documented history of use spanning over 2500 years [1]. Today, lavender essential oil is widely utilized in pharmacology, cosmetics and the food industry, with the global lavender oil market valued at USD 530.5 million in 2020 [2,3]. The essential oil’s chemical composition is dominated by monoterpenoids, primarily linalool, linalyl acetate, borneol and camphor; however, their relative abundances vary significantly depending on geographic origin and cultivar-specific genetic traits [2,3]. Over centuries of cultivation, numerous landraces and commercial varieties have been selected for specific oil profiles and adaptation to local climates, making the genus a rich subject for phytochemical and genetic studies. Alongside pure *L. angustifolia* forms, interspecific hybrids such as lavandin (*Lavandula* × *intermedia*) are widely grown for essential oil production, yet their genetic identity and relationships remain insufficiently characterized at the molecular level.

Despite its economic importance, the molecular-genetic foundation of the genus *Lavandula* has long remained underdeveloped. A pivotal advance was the publication of a chromosome-scale nuclear genome for *L. angustifolia*, which revealed gene duplications associated with terpenoid biosynthesis [1]. However, for phylogenetic and population studies, chloroplast genomes are equally critical. The angiosperm chloroplast genome has a compact size (150–160 kbp) and a conserved quadripartite structure; predominantly maternal transmission makes it a valuable system for tracing the evolutionary lineage history of plastids [4,5].

Chloroplast genomes are now widely used in plant systematics and DNA barcoding. Since the first chloroplast genome was sequenced [6], advances in sequencing technologies have made it possible to obtain complete chloroplast genomes for many species [7]. Short barcoding regions such as *matK*, *rbcL*, *psbA-trnH*, and *trnL-trnF* were initially used for species identification, but they often lack resolution for closely related cultivars [8]. Complete chloroplast genomes provide much higher phylogenetic resolution and have been successfully applied to study cultivar origins in grapevine, date palm, rice, and tomato [9,10,11,12]. For *Lavandula*, this approach remains largely underexplored. Although several complete chloroplast genome sequences from individual taxa have recently been deposited in public repositories [13,14,15,16], comprehensive comparative data within the genus remain very limited. In particular, there is an absence of comparative chloroplast genomic studies evaluating commercially cultivated landraces alongside interspecific hybrids, which hampers fine-scale evolutionary analyses and the development of reliable candidate markers for cultivar identification.

In this study, we aimed to fill this gap by obtaining complete chloroplast genome sequences for six *Lavandula* accessions (four *L. angustifolia* and two *L.* × *intermedia*) using de novo assembly from whole-genome shotgun data. These specific accessions were selected because they represent economically prominent cultivars and commercial hybrids widely cultivated for essential oil production in regional agriculture; although their horticultural traits are well established, their complete chloroplast genomes are newly reported and characterized here for the first time. The specific objectives were: (i) to assemble and annotate the six chloroplast genomes, characterizing their size, GC-content, gene content, and boundaries between the LSC, SSC and IR regions; (ii) to identify and compare simple sequence repeats (SSRs) and long repeats across the genomes; (iii) to evaluate nucleotide diversity and codon usage patterns to detect variable regions that could serve as potential molecular markers; and (iv) to reconstruct a phylogenetic tree based on whole chloroplast genome alignments, placing the new *Lavandula* genomes within the family Lamiaceae and clarifying their evolutionary relationships within this taxonomically complex group. The resulting data set will not only expand the reference chloroplast genome collection for *Lavandula* but also provide a solid foundation for future population genetics, phylogeography and DNA-based authentication of lavender cultivars.

## 2. Results

### 2.1. Assembly and General Characteristics of Lavandula Chloroplast Genomes

Complete chloroplast genome sequences were obtained for six *Lavandula* accessions (four *L. angustifolia* and two *L.* × *intermedia*) via de novo assembly from whole-genome shotgun data. The assembled genomes ranged from 151,431 to 151,480 bp, with a maximum variation of 49 bp. All genomes displayed the following canonical quadripartite structure: a large single-copy region (LSC) of 82,682–82,837 bp, a small single-copy region (SSC) of 17,579–17,580 bp, and two inverted repeats (IRa and IRb) of 25,532–25,586 bp each. The GC content was uniform across samples, varying from 37.7% to 37.8% (Table 1).

Gene content was identical among the six genomes, comprising 113 genes: 79 protein-coding genes, 30 tRNA genes, and 4 rRNA genes. Duplicated genes within the IR regions included five protein-coding genes (*ndhB*, *rpl2*, *rpl23*, *rps7*, *ycf2*), seven tRNA genes, and four rRNA genes. Eleven protein-coding genes and eight tRNA genes contained a single intron, whereas *clpP1* possessed two introns. No gene losses, pseudogenisations, or major rearrangements were detected.

**Figure 1:**
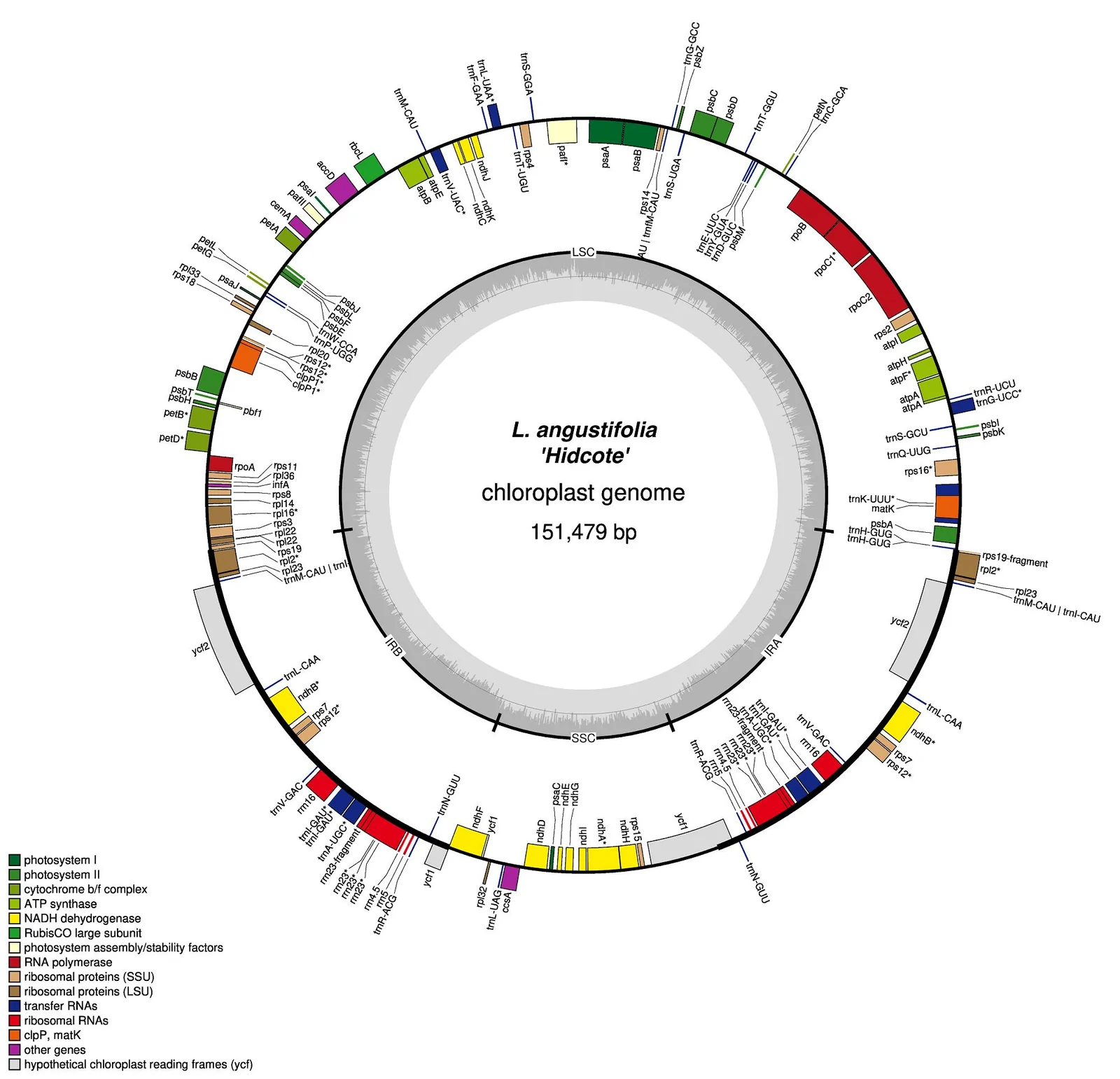
Circular map of the *Lavandula angustifolia* ‘Hidcote’ chloroplast genome. Genes shown outside the circle are transcribed clockwise; those inside are transcribed counterclockwise. Colours indicate functional gene groups: photosynthesis (dark green), transcription/translation (light green), ribosomal proteins (orange), tRNA genes (blue), rRNA genes (red), and conserved open reading frames (grey). The dark grey histogram in the inner circle represents the GC content. The inner circle also marks the boundaries of the large single-copy (LSC), small single-copy (SSC), and inverted repeat (IRa/IRb) regions. Maps for the remaining five accessions are provided in Supplementary Figures S1–S5.

Circular genome maps confirmed the conserved gene order and transcriptional orientation across all samples. A representative map for *L. angustifolia* ‘Hidcote’ is shown in Figure 1; maps for the remaining five accessions are provided in Supplementary Figures S1–S5.

### 2.2. Boundary Dynamics of Structural Regions

**Figure 2:**
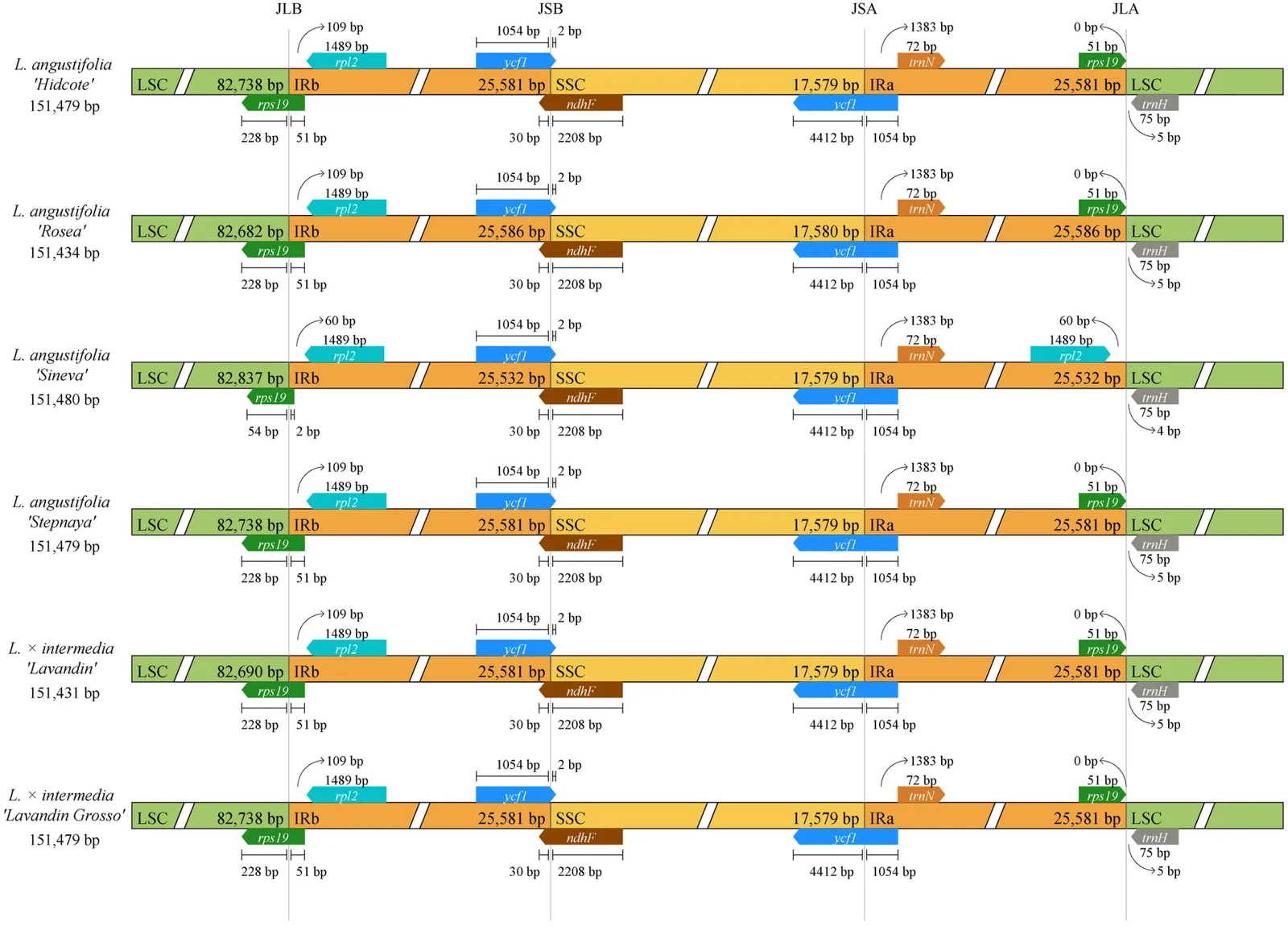
Comparison of the LSC, SSC and IR boundaries in the chloroplast genomes of six *Lavandula* accessions. The diagram shows the junctions between the LSC/IRb (left), IRb/SSC (center), and SSC/IRa (right) regions. Genes and their orientations are indicated by arrows; distances (in bp) from the boundary to the nearest gene are shown. The *rps19* gene is partially or fully located in the LSC depending on the accession, while the *ycf1* gene consistently spans the IRb/SSC and SSC/IRa junctions. The *trnH* gene is located at the IRa/LSC boundary.

The junctions between the LSC, IR, and SSC regions were analyzed to detect potential expansions or contractions (Figure 2). At the LSC/IRb boundary (JLB), the *rps19* gene was positioned across the boundary with 228 bp (or 54 bp in ‘Sineva’) in the LSC and 51 bp (2 bp in ‘Sineva’) extending into IRb, located adjacent to *rpl2* (1489 bp in length, situated 109 bp or 60 bp from the junction). At the IRb/SSC junction (JSB), the *ycf1* gene extended 1054 bp into IRb, while *ndhF* (2208 bp) was located in the SSC region with a 2 bp overlap/distance at the JSB boundary and 30 bp distance to IRb. The SSC/IRa junction (JSA) was positioned within *ycf1*, spanning 4412 bp in the SSC and 1054 bp into IRa, adjacent to *trnN* (72 bp, located 1383 bp from JSA). The IRa/LSC junction (JLA) spanned near *trnH* (75 bp), which was located in the LSC region at 5 bp (or 4 bp in ‘Sineva’) from the JLA boundary, opposite *rps19* (51 bp in IRa, or *rpl2* at 60 bp in ‘Sineva’). These boundary positions and gene distances were highly conserved across the accessions, confirming that no major structural rearrangements occurred.

### 2.3. Sequence Conservation and Nucleotide Diversity

**Figure 3:**
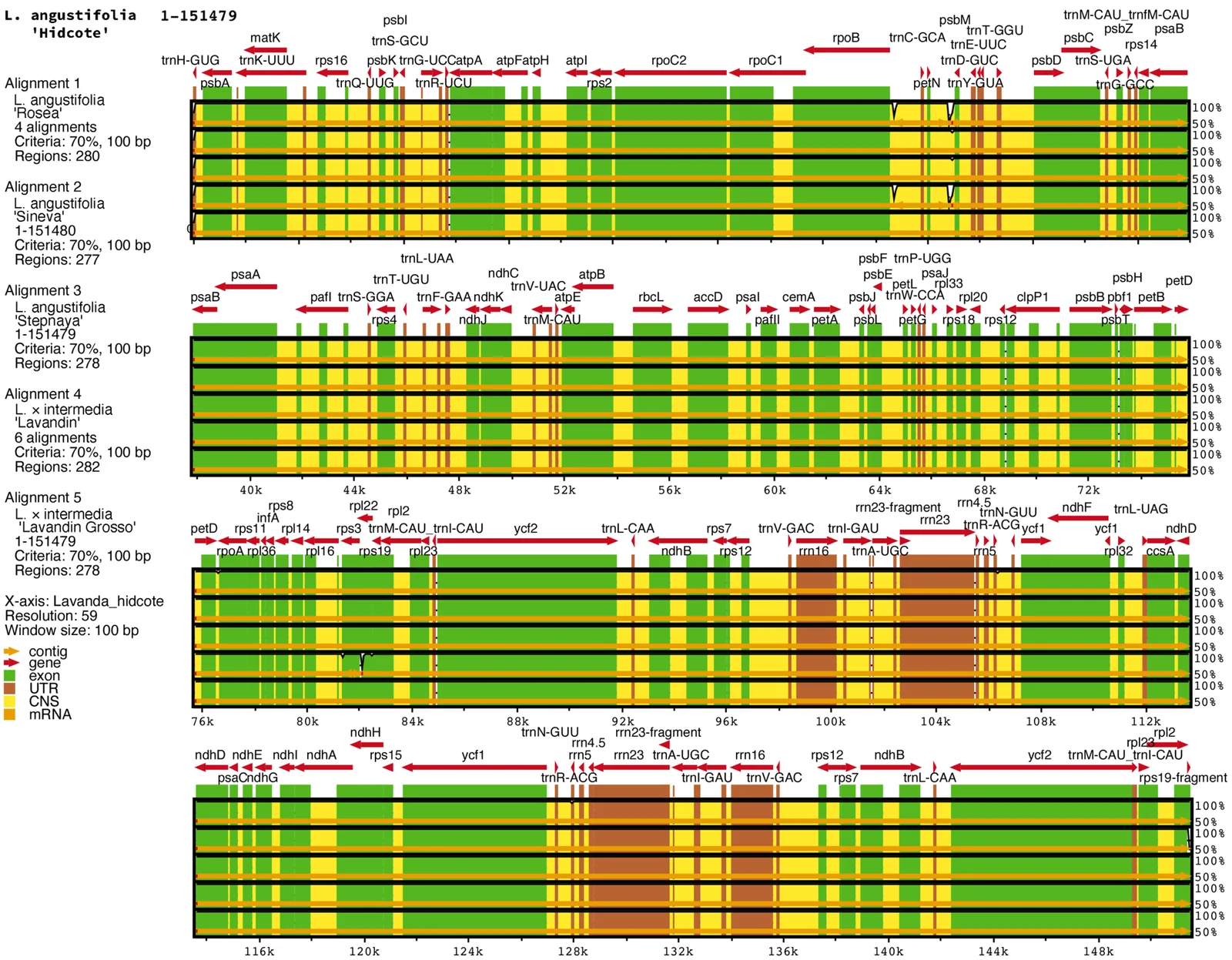
mVISTA sequence identity plot of six *Lavandula* chloroplast genomes using *L. angustifolia* ‘Hidcote’ as a reference. The x-axis shows the genomic coordinates of the reference; the y-axis displays the percentage of identity (50–100%). Coloured bars indicate annotated features: exons (green), UTR (brown), CNS (orange), and mRNA (yellow). Peaks of reduced identity correspond to non-coding regions, while protein-coding exons are highly conserved.

Whole-genome alignment using mVISTA with *L. angustifolia* ‘Hidcote’ as a reference (Figure 3) demonstrated a high degree of collinearity and sequence identity. The alignment was performed with a 70% identity threshold and a 100 bp window. The number of conserved regions ranged from 277 (*L. angustifolia* ‘Sineva’) to 282 (*L.* × *intermedia* ‘Lavandin’), indicating that the genomes were almost entirely identical. Protein-coding exons (blue) showed the highest conservation, while intergenic regions (grey) exhibited occasional reductions in identity. No inversions or translocations were detected.

**Figure 4:**
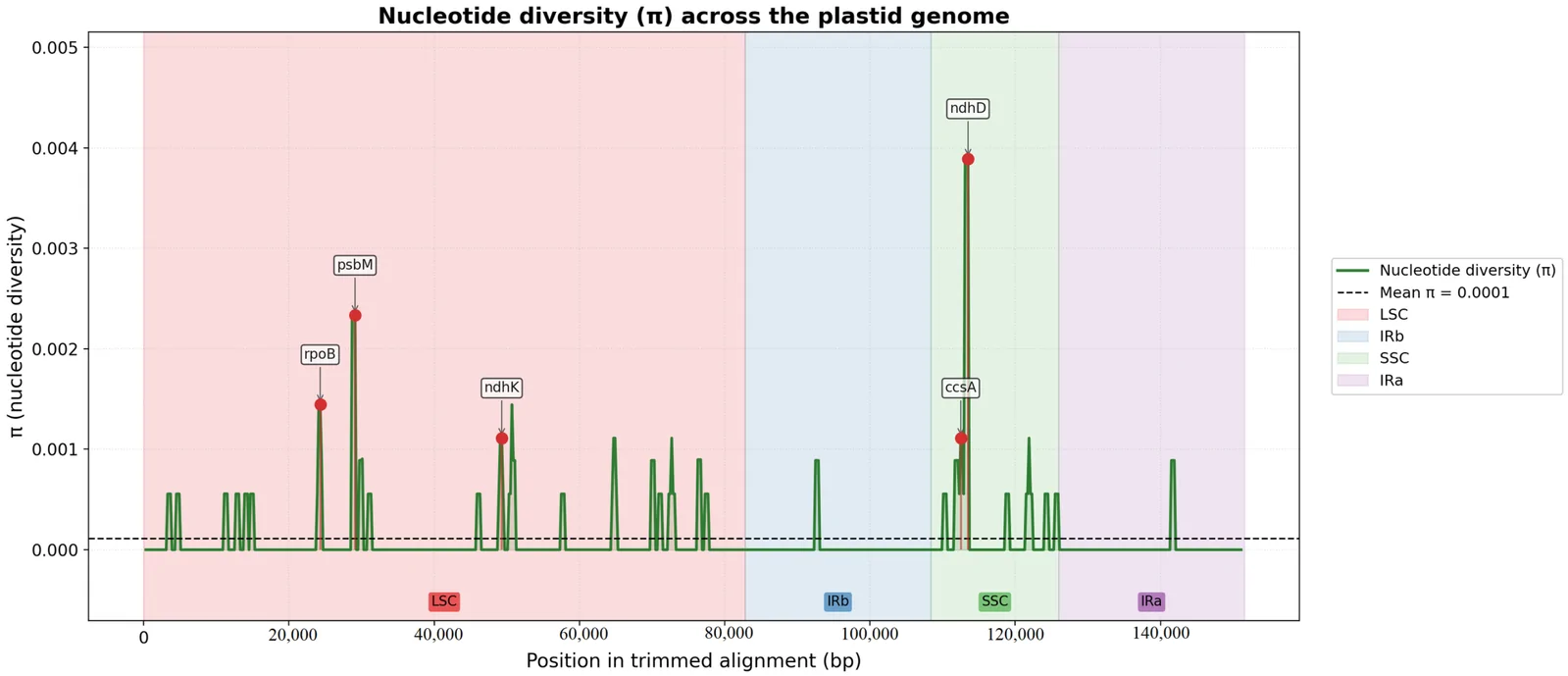
Nucleotide diversity (π) profile of the six *Lavandula* chloroplast genomes. Values were calculated in sliding windows of 600 bp with a step size of 200 bp. The dashed horizontal line indicates the mean π across the entire alignment (π ≈ 0.0001). Vertical lines mark the boundaries between the LSC, IRb, SSC and IRa regions. Elevated π values are observed in several gene regions (*rpoB*, *psbM*, *ndhK*, *ccsA*, and *ndhD*).

Nucleotide diversity (π) was calculated in sliding windows of 600 bp with a step size of 200 bp (Figure 4). The mean π across the entire genome was 0.0001, as indicated by the dashed horizontal line. Most windows showed π values close to zero, reflecting extremely low divergence. However, several loci exhibited distinct peaks of elevated diversity, including gene regions (*rpoB*, *psbM*, *ndhK*, *ccsA*, and *ndhD*). The highest peak, reaching approximately 0.0039–0.004, was observed in the *ndhD* gene region. These variable regions represent preliminary candidate loci that warrant further validation on larger and more diverse population samples to test their utility as potential molecular markers.

### 2.4. Identification of Simple Sequence Repeats

**Figure 5:**
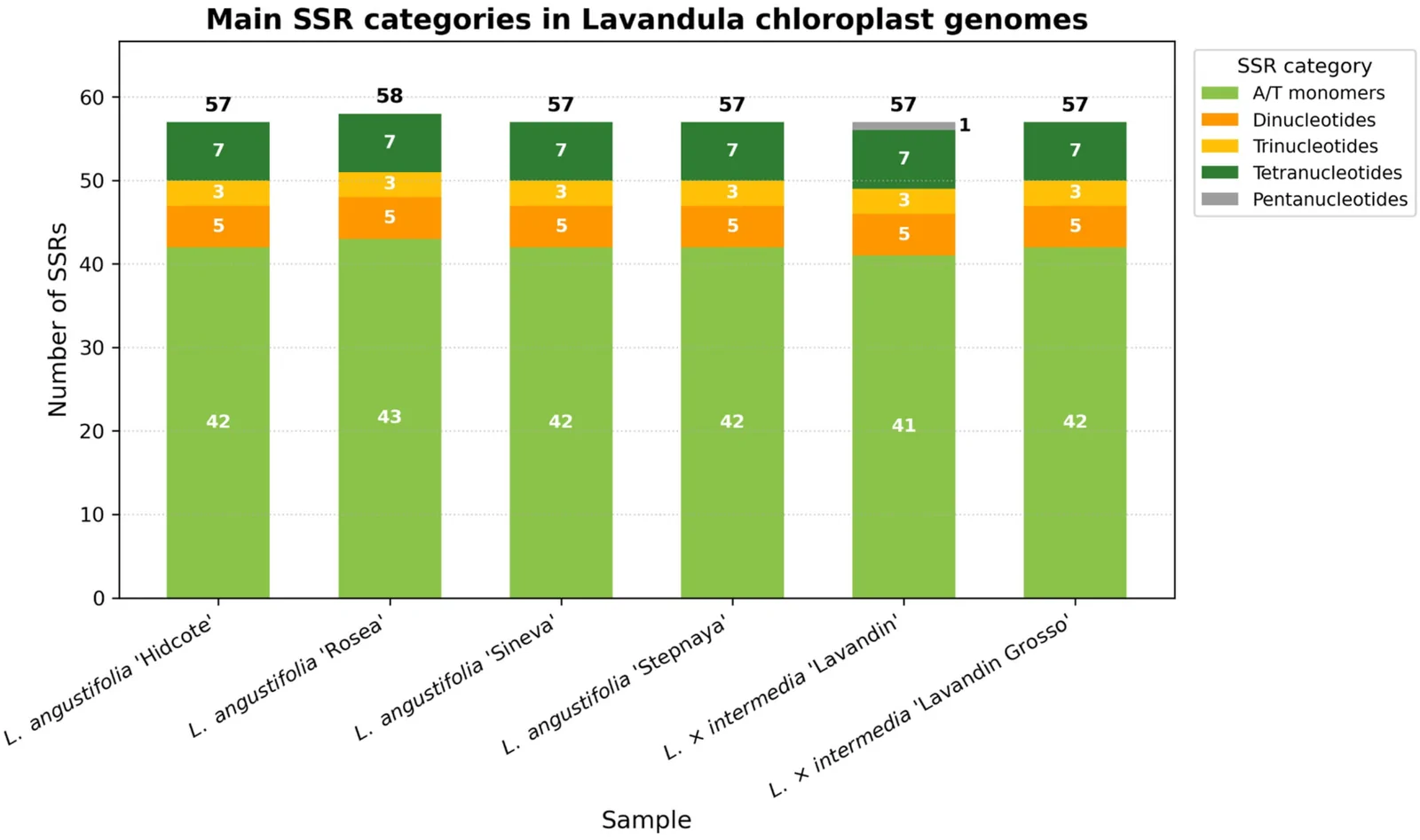
Number and types of simple sequence repeats (SSRs) detected in the six *Lavandula* chloroplast genomes. SSRs are classified by repeat unit: monomers, dimers, trimers, tetramers and pentamers. The number of SSRs per sample ranges from 57 to 58, with monomers being the most abundant (41–43 per genome). SSR profiles are highly similar across all accessions.

Simple sequence repeats (SSRs) were identified and classified into six categories: monomers, dimers, trimers, tetramers, pentamers, and hexamers (Figure 5, Supplementary Table S1). The total number of SSRs per genome was highly consistent: five of six accessions contained exactly 57 SSRs, while *L. angustifolia* ‘Rosea’ contained 58. The distribution across categories was nearly identical for all samples: monomers (41–43), dimers (exactly 5), trimers (exactly 3) and tetramers (exactly 7). Pentamers were present only in *L.* × *intermedia* ‘Lavandin’ (1 locus), while hexamers were absent across all genomes. This uniformity indicates that SSR content is a stable genomic feature within the studied group and does not distinguish between *L. angustifolia* and *L.* × *intermedia* accessions.

### 2.5. Analysis of Synonymous Codon Usage

For protein-coding genes, the relative synonymous codon usage (RSCU) metric was calculated [17]. It shows how often a specific synonymous codon is used compared to the expected frequency under uniform usage of all codons encoding the same amino acid [17]. The heatmap of initial RSCU values shows that the codon usage profiles of all six samples are practically identical (Supplementary Figure S6). RSCU values ranged from approximately 0.36 to 1.9. Codons with the highest RSCU values are located at the top of the heatmap, and codons with lower values are at the bottom. The chloroplast genomes of the studied samples are characterized by a bias towards codons ending in A or T. This pattern is consistent with the AT richness of chloroplast DNA and general codon usage features [18]. The RSCU analysis showed that the codon usage profiles of the six *Lavandula* chloroplast genomes are virtually indistinguishable. This is consistent with the high conservation of protein-coding sequences. No pronounced changes associated with adaptation or random shift in codon composition were detected in the studied group.

### 2.6. Ka/Ks Ratio Analysis

Additionally, the ratio of nonsynonymous to synonymous substitution rates (Ka/Ks) was evaluated for the set of protein-coding genes to assess selective pressure [19,20]. As standard, Ka/Ks values < 1 indicate purifying (negative) selection, values around 1 correspond to neutral evolution, and Ka/Ks > 1 suggests potential positive (diversifying) selection [19]. On the scatter plot (Supplementary Figure S7), most data points were located below the Ka = Ks line, corresponding to Ka < Ks, which aligns with the overall structural and functional conservatism of chloroplast genes [21]. While certain genes (e.g., *rpl33* and *ycf2*) displayed elevated Ka/Ks values, extreme caution is required when interpreting these observations. Due to the extremely close phylogenetic proximity of the accessions and the near-zero number of synonymous substitutions (ultra-low Ks), the Ka/Ks ratio becomes highly sensitive to numerical instability and stochastic noise [22]. Consequently, these estimations should be viewed strictly as preliminary descriptive metrics rather than conclusive evidence of positive selection.

### 2.7. Identification of Long Repeats

**Figure 6:**
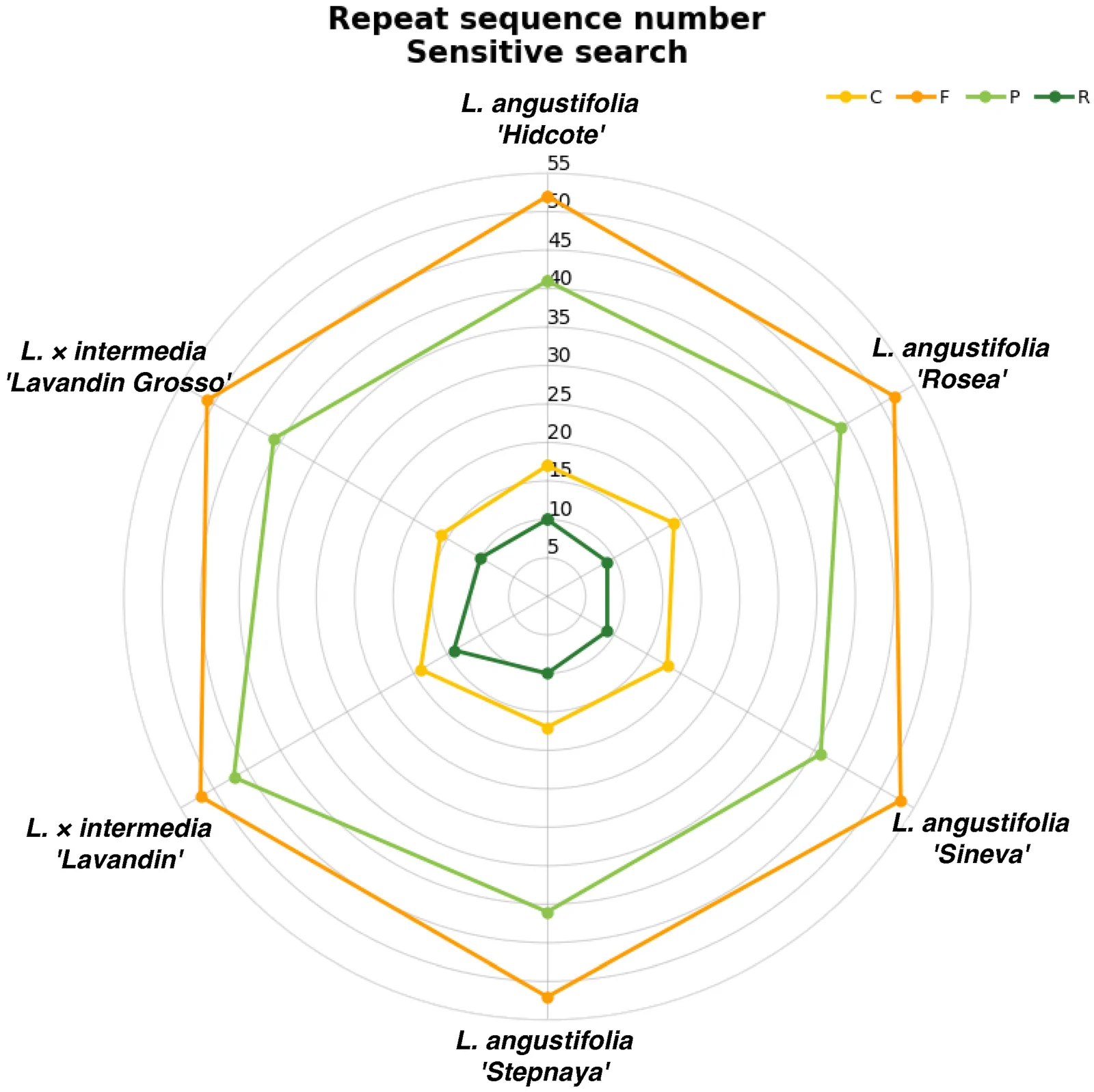
Distribution of long repeat sequences in the six *Lavandula* chloroplast genomes. Repeats are categorised as forward (F), reverse (R), complementary (C), and palindromic (P). Parameters: length ≥ 20 bp, identity ≥ 80%, mismatches ≤ 4, gaps not allowed, and overlap permitted. The total number of repeats ranges from 112 to 118 per genome. Hybrid accessions (‘Lavandin’, ‘Lavandin Grosso’) show higher numbers of forward and palindromic repeats compared to pure *L. angustifolia* accessions. Maximum repeat lengths: P = 25,586 bp, F = 56 bp, C = 29 bp, and R = 28 bp.

Long repetitive sequences were identified using a sensitive search approach with the following parameters: repeat length ≥ 20 bp, sequence identity ≥ 80%, mismatches ≤ 4, gaps not allowed, and overlapping repeats permitted. Four types of repeats were analysed: forward (F), reverse (R), complementary (C), and palindromic (P) repeats (Figure 6).

The total number of repeats varied among accessions. The highest total counts were observed in hybrid samples: *Lavandula* × *intermedia* ‘Lavandin’ (118 repeats: F = 54, P = 41, C = 16, R = 7) and *Lavandula* × *intermedia* ‘Lavandin Grosso’ (118 repeats: F = 55, P = 41, C = 15, R = 7). The lowest counts were found in *L. angustifolia* ‘Hidcote’ (112 repeats: F = 51, P = 39, C = 15, R = 7). Intermediate values were observed in *L. angustifolia* ‘Rosea’ (113 repeats: F = 53, P = 38, C = 15, R = 7), *L. angustifolia* ‘Sineva’ (116 repeats: F = 53, P = 41, C = 15, R = 7), and *L. angustifolia* ‘Stepnaya’ (117 repeats: F = 54, P = 40, C = 15, R = 8).

Forward repeats (F) were the most abundant type across all accessions, ranging from 51 (‘Hidcote’) to 55 (‘Lavandin Grosso’). Palindromic repeats (P) were the second most frequent, ranging from 38 (‘Rosea’) to 41 (‘Sineva’, ‘Lavandin’, ‘Lavandin Grosso’). Complementary repeats (C) showed remarkable stability, with 15 repeats in all accessions except ‘Lavandin’ (16). Reverse repeats (R) were the least abundant, with 7 repeats in all accessions except *L. angustifolia* ‘Stepnaya’ (8).

The maximum repeat lengths differed substantially by type. Palindromic repeats (P) exhibited the greatest maximum length at 25,586 bp, which is comparable to the size of the inverted repeat (IR) regions. Forward repeats reached a maximum of 56 bp, complementary repeats reached 29 bp, and reverse repeats reached 28 bp. The exceptionally long palindromic repeats correspond to the large, inverted repeats (IRa and IRb) characteristic of angiosperm chloroplast genomes.

Notably, hybrid accessions (‘Lavandin’ and ‘Lavandin Grosso’) consistently displayed higher numbers of forward and palindromic repeats compared to the pure *L. angustifolia* accessions. This suggests that hybridization may be associated with an accumulation of long repetitive elements in the chloroplast genome. The stability of complementary and reverse repeat counts across all samples indicates that these repeat types are highly conserved within the genus.

### 2.8. Phylogenetic Analysis

**Figure 7:**
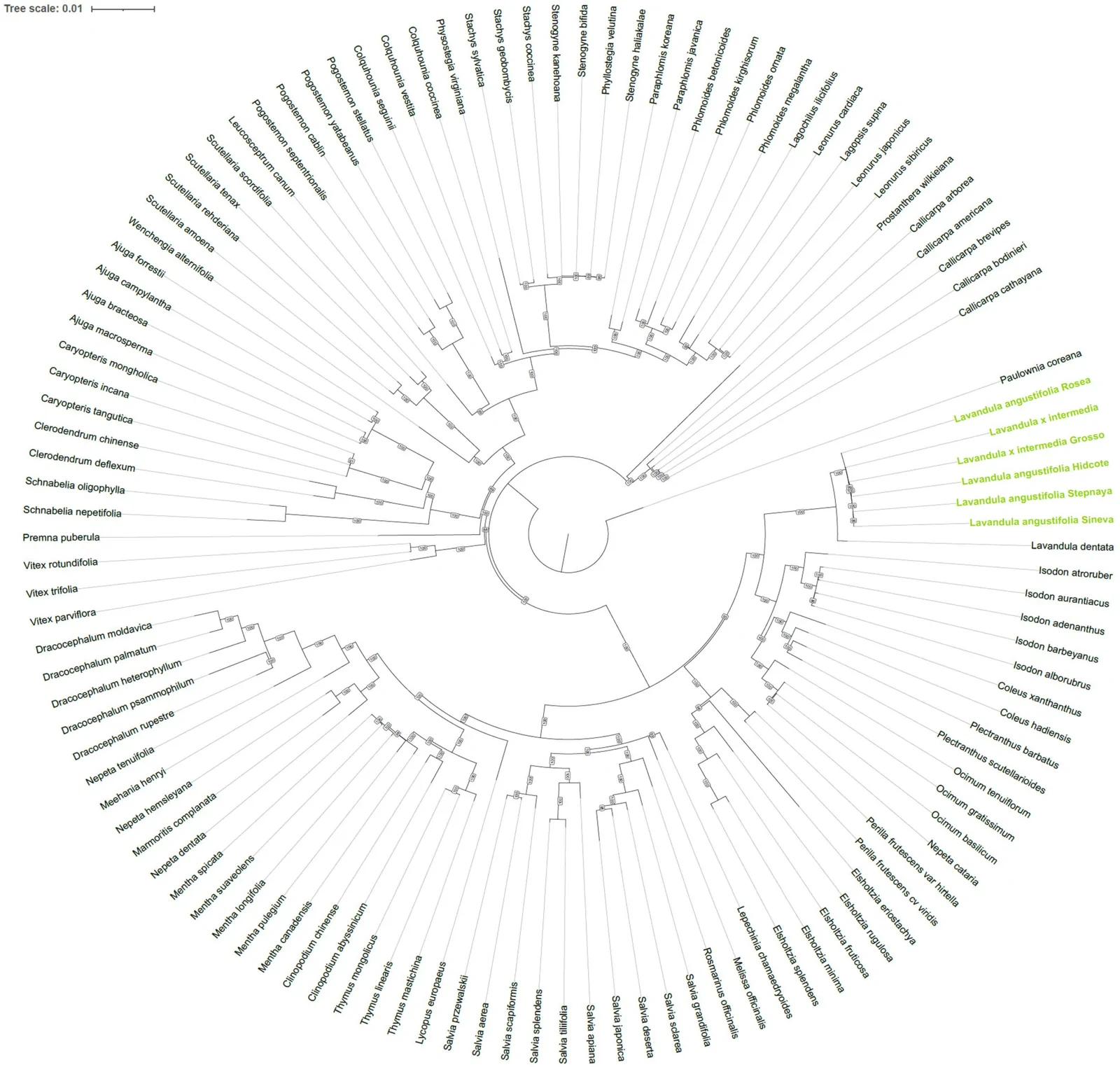
Maximum-likelihood (ML) phylogenetic tree of Lamiaceae based on whole chloroplast genome alignments, rooted using *Paulownia coreana* as an outgroup. The tree includes the six newly assembled *Lavandula* genomes (highlighted) and a broad representation of Lamiaceae taxa. Bootstrap support values are indicated as percentages at the nodes. Topological congruency was fully confirmed by Bayesian Inference (BI), which yielded an identical tree topology (Robinson–Foulds distance, RF = 0); the corresponding BI tree and tanglegram are presented in Supplementary File S1.

A maximum likelihood phylogenetic tree was reconstructed based on whole chloroplast genome alignments (Figure 7). The tree included a broad representation of Lamiaceae, with bootstrap support values indicated as percentages at the nodes. The BI-derived phylogenetic tree showed no significant differences (Supplementary File S1) compared to ML. Both ML and BI methods yielded identical topologies (Robinson–Foulds distance, RF = 0), with the only node having posterior probability PP < 0.95 being (*Caryopteris incana*/*C. mongholica*) with PP = 0.73, and *Paulownia coreana* was used as an outgroup in both methods. The genus *Lavandula* formed a monophyletic group, positioned within the subfamily Nepetoideae. The six newly assembled accessions (*Lavandula angustifolia* ‘Hidcote’, *Lavandula angustifolia* ‘Rosea’, *Lavandula angustifolia* ‘Sineva’, *Lavandula angustifolia* ‘Stepnaya’, *Lavandula* × *intermedia* ‘Lavandin’, and *Lavandula* × *intermedia* ‘Lavandin Grosso’) clustered together with *Lavandula angustifolia* and *Lavandula* × *intermedia* from public databases.

In the broader family context, *Lavandula* was placed in close proximity to *Salvia* (e.g., *Salvia miltiorrhiza*, *Salvia officinalis*) and *Elsholtzia*, while remaining distinct from *Mentha*, *Rosmarinus*, and *Ocimum*. This topology is consistent with the current taxonomic classification of Lamiaceae, where *Lavandula*, *Salvia*, and *Elsholtzia* belong to the same subfamily Nepetoideae but to different tribes (Ocimeae and Mentheae, respectively). The hybrid accessions *Lavandula* × *intermedia* ‘Lavandin’ and *Lavandula* × *intermedia* ‘Lavandin Grosso’ were positioned adjacent to the *L. angustifolia* cluster, consistent with the maternal origin of *L. angustifolia*.

## 3. Discussion

The chloroplast genome is an indispensable tool for evolutionary biology, phylogenomics, and germplasm characterization due to its maternal inheritance, conserved structure, and low rate of nucleotide substitution [4,5,23]. In this study, we performed de novo assembly and comprehensive annotation of six complete chloroplast genomes from four economically important cultivars of *Lavandula angustifolia* (‘Hidcote’, ‘Rosea’, ‘Sineva’, ‘Stepnaya’) and two interspecific hybrids of *Lavandula* × *intermedia* (‘Lavandin’, ‘Lavandin Grosso’).

RSCU analysis demonstrated nearly identical codon usage profiles across all six accessions, characterized by a distinct bias towards AT-ending codons, consistent with plastid DNA AT-richness. Evaluation of the Ka/Ks ratio similarly pointed toward general purifying selection across coding regions; however, given the minimal sequence divergence among closely related accessions and potential numerical artifacts at ultra-low Ks values, inferences regarding site-specific positive selection remain highly speculative and require verification across broader taxonomic sampling.

Recent comparative chloroplast genomic investigations across the family Lamiaceae have further emphasized the evolutionary stability and diagnostic potential of complete chloroplast genomes. In particular, the recently characterized chloroplast genome of *Lavandula maroccana* [16] revealed a conserved quadripartite architecture and gene content congruent with our findings in *L. angustifolia* and *L.* × *intermedia*, while exhibiting taxon-specific divergence patterns in localized regions. Similar patterns of lineage-specific divergence hotspots alongside highly conserved inverted repeat boundaries have been reported in other closely related Nepetoideae genera, including *Salvia*, *Thymus*, and *Scutellaria* [24,25,26], corroborating that while the core plastid sequences remain constrained by purifying selection, identified polymorphic coding and boundary loci provide primary candidate targets for fine-scale phylogeographic analysis and taxonomic discrimination.

The assembled genomes exhibited a highly conserved quadripartite structure, with sizes ranging from 151,431 to 151,480 bp and an identical repertoire of 113 genes (79 protein-coding, 30 tRNA, and 4 rRNA genes). The structural boundaries across the LSC, SSC, and IR junctions showed minimal shifts, with *rps19* and *ycf1* spanning the junctions in a stable configuration across all accessions [27]. The absence of gene losses, pseudogenization events, or structural rearrangements confirms the profound evolutionary stability of the chloroplast genome across both pure lavender cultivars and cultivated hybrids [1,5].

Despite the overall high sequence identity, sliding-window nucleotide diversity analysis revealed distinct divergence hotspots, including *ndhD*, *ccsA*, *rpoB*, *psbM*, and *ndhK*. Standard DNA barcoding regions (e.g., *matK*, *rbcL*, and *trnH-psbA*) frequently fail to provide sufficient resolution to discriminate between closely related cultivars or complex hybrid accessions [28]. The polymorphic loci identified in our chloroplast genomes represent promising candidate regions that require experimental evaluation across representative population collections to confirm whether they can serve as reliable molecular markers for species or cultivar identification [2,9].

Analysis of repetitive elements demonstrated uniform SSR profiles (56–57 loci dominated by A/T mononucleotides), reflecting genomic stability [29]. In contrast, long repeat analysis uncovered a higher count of forward and palindromic repeats in the hybrid accessions (*L.* × *intermedia*) compared to pure *L. angustifolia*. This accumulation of repeat sequences suggests subtle structural dynamics following interspecific hybridization events.

Phylogenomic reconstruction based on whole-chloroplast genome sequences placed the genus *Lavandula* as a monophyletic lineage within the subfamily Nepetoideae, closely related to *Salvia* and *Elsholtzia* [30]. Crucially, both *L.* × *intermedia* accessions nested directly within the *L. angustifolia* clade, confirming that *L. angustifolia* served as the maternal parent in the genesis of these commercially cultivated lavandins [31]. However, while chloroplast genome data effectively trace maternal ancestry, they reflect only uniparental inheritance and cannot independently reconstruct the full hybridization event without biparental nuclear genomic markers. Together, these six newly assembled genomes provide a foundational dataset that bridges fundamental chloroplast evolution with applied breeding, cultivar protection, and quality control in the lavender industry.

## 4. Materials and Methods

### 4.1. Plant Materials, DNA Extraction, and Sequencing

Plant material for this study was collected from the commercial lavender plantation “Maison Des Plantes Aromatiques”, located at the 26th km of the A-290 highway, Novorossiysk, Krasnodar Krai, Russia (44°42′ N, 37°46′ E). Six *Lavandula* accessions were analyzed: four *L. angustifolia* (‘Hidcote’, ‘Rosea’, ‘Sineva’, ‘Stepnaya’) and two interspecific hybrids *Lavandula* × *intermedia* (‘Lavandin’, ‘Lavandin Grosso’). The plant material was collected in 2024 during the flowering season. Representative voucher herbarium specimens were prepared from the collected materials and are deposited at the National Research Center “Kurchatov Institute” under internal collection identifiers (voucher IDs: NRCKI-LAV-01 to NRCKI-LAV-06; photographic documentation provided in Supplementary Figures S8–S13). Taxonomic identity was confirmed by botanists during the collection and pre-processing stages based on standard diagnostic morphological keys.

Total genomic DNA was extracted using a modified CTAB (cetyltrimethylammonium bromide) protocol [32,33]. The concentration and quality of extracted DNA were assessed using a Nanodrop 1000 spectrophotometer (Thermo Fisher Scientific, Waltham, MA, USA) and a Qubit 4.0 fluorometer with the Qubit™ dsDNA BR Assay Kit (Thermo Fisher Scientific, Waltham, MA, USA). DNA fragment libraries were prepared for sequencing using the NEBNext^®^ Ultra™ II DNA Library Prep Kit for Illumina^®^ (New England BioLabs, Ipswich, MA, USA) according to the manufacturer’s protocol. The resulting libraries were sequenced on a SurfSeq 5000^TM^ platform (GeneMind, Shenzhen, China) using 2 × 150 bp paired-end chemistry.

### 4.2. Genome Assembly and Annotation

Raw sequencing reads were processed using fastp v0.23.4 [34] to remove adapter sequences and low-quality reads. Quality filtering was performed with the following parameters: automatic adapter detection (--detect_adapter_for_pe), 5′ and 3′ trimming of low-quality bases (--cut_front, --cut_tail), a mean quality threshold of Q20 (--cut_mean_quality 20), a minimum read length of 50 bp after trimming (--length_required 50), and a maximum of five ambiguous N bases per read (--n_base_limit 5).

Chloroplast genomes were assembled de novo using GetOrganelle v1.7.7.0 [35] in plant chloroplast genome assembly mode (-F embplant_pt). For each sample, a k-mer set of 21, 45, 65, 85, 105, and 115 was used. For more complex assemblies, an extended k-mer set of 21, 33, 45, 57, 69, 81, 93, 105, 115, and 127 was applied. Coverage was limited using the parameters --reduce-reads-for-coverage 300 and --max-reads 20,000,000. For samples where GetOrganelle did not recover a complete circular path, NOVOPlasty v4.3.1 [36] was employed with the intermediate contig as a seed sequence, using the chloro mode and an expected genome size of 150–155 kbp.

Graph-based assembly validation was performed using BandageNG v0.9.0 [37] to visualize the de Bruijn graphs and resolve ambiguities in the IR regions.

To calculate the mean sequencing depth for each assembled chloroplast genome and separately for the inverted repeat (IR) regions, clean reads were mapped back to the corresponding complete chloroplast genome assemblies using BWA-MEM v0.7.17 [38]. Alignment files were processed, sorted, and indexed using samtools v1.17 [39]. The average coverage depth across the entire chloroplast genome and within the annotated IR regions was subsequently calculated using the samtools depth command.

Annotation of the assembled genomes was conducted using the GeSeq web server [40] with default parameters. tRNA genes were identified using tRNAscan-SE v2.0.12 [41] with default settings. rRNA genes were annotated using BLASTN v2.12.0 [42] against the NCBI nucleotide database. Circular maps of the chloroplast genomes were visualized using OGDRAW v1.3.1 [43]. All raw sequencing data generated in this study have been deposited in the NCBI Sequence Read Archive (SRA). The BioProject under number PRJNA1517218 contains raw sequencing data for four cultivars of *L. angustifolia* (‘Hidcote’, ‘Rosea’, ‘Sineva’, ‘Stepnaya’) and two hybrids of *Lavandula* × *intermedia* (‘Lavandin’, ‘Lavandin Grosso’).

### 4.3. Comparative Genome Analysis

The six complete *Lavandula* chloroplast genomes were compared using mVISTA [44] with the shuffle-LAGAN alignment model, using *L. angustifolia* ‘Hidcote’ as the reference sequence. The analysis was performed with a 70% identity threshold and a 100 bp window size. IRscope [27] was used to visualize and compare the boundaries between the LSC, IR, and SSC regions across all six genomes.

For nucleotide diversity analysis, the six genome sequences were cleaned by removing all non-canonical nucleotide characters (excluding A, T, G, C, and N) and replacing them with N. The cleaned sequences were then aligned using MAFFT v7.427 [45] with the --auto parameter. Following alignment, columns with less than 10% non-gap characters were removed (i.e., only positions with at least 10% of sequences having a nucleotide were retained). Nucleotide diversity (π) was calculated using a custom Python 3.14.0 script with sliding windows of 600 bp and a step size of 200 bp. For each window, π was computed as the average pairwise nucleotide difference across all six sequences. The boundaries of the LSC, IRb, SSC, and IRa regions were mapped onto the trimmed alignment using the reference genome *L. angustifolia* ‘Stepnaya’ as a coordinate anchor. Gene annotations were loaded from the GFF3 file of the ‘Stepnaya’ genome to identify gene locations in the alignment. The top five peaks of nucleotide diversity located within annotated genes were automatically identified and labelled on the plot.

### 4.4. Analysis of the Chloroplast Genome for Repetitive Sequences

Simple sequence repeats (SSRs) were identified in the six *Lavandula* chloroplast genomes using MISA v2.1 software [29]. The minimum repeat unit thresholds were configured as follows: 10 for mononucleotide SSRs, 5 for dinucleotide SSRs, 4 for trinucleotide SSRs, and 3 for tetranucleotide, pentanucleotide, and hexanucleotide SSRs. For visualization, SSRs were grouped into four categories: A/T monomers, AT/TA dimers, other trimers, and tetramers. The four most informative categories were plotted as stacked bar charts using a custom Python script with the Matplotlib 3.9.0 library.

Long repeated sequences were identified using a custom Python script. The algorithm implemented a search for duplicated segments based on string matching, generating four search modes for each genome to identify forward (F), reverse (R), complementary (C), and palindromic (P) repeats. The search parameters were: a minimum repeat length of 20 bp, a minimum sequence identity of 80%, up to 4 mismatches allowed, no gaps permitted, and overlapping repeats allowed. This set of parameters corresponds to the sensitive search mode used in the analysis. For each repeat type, the total number of repeats and the maximum repeat length were recorded for each genome. The results were visualized as a radar (spider) chart.

### 4.5. Codon Usage Analysis

Codon usage bias was analysed for the 79 protein-coding genes extracted from the annotated GenBank files. For each genome, coding sequences (CDSs) were extracted and translated using the standard genetic code for plastids (NCBI genetic code 11) as implemented in the Biopython library [46]. Stop codons and codons containing ambiguous nucleotides were excluded from the analysis.

Relative synonymous codon usage (RSCU) was calculated for each codon as the ratio of the observed frequency to the expected frequency under uniform usage of all synonymous codons. RSCU values greater than 1 indicate preferential codon usage, values equal to 1 indicate no bias, and values less than 1 indicate under-representation. A centered RSCU matrix was generated by subtracting the mean RSCU value for each codon across all samples from the individual sample values. Heatmaps of both raw and centered RSCU values were generated using the Seaborn 0.3.12 and Matplotlib libraries.

### 4.6. Selection Pressure Analysis

To evaluate selective pressure on the protein-coding genes, the ratio of nonsynonymous (Ka) to synonymous (Ks) substitution rates was calculated for the 79 shared protein-coding genes across the six *Lavandula* genomes. Ka and Ks values were computed using KaKs_Calculator 3.0 [47] with the ParaAT v2.0 [48] wrapper for automated generation of codon-aligned sequences. Results with excessively high Ka values, likely due to extremely low Ks values, were excluded from further interpretation. A Ka/Ks ratio greater than 1 indicates positive selection; a ratio equal to 1 indicates neutral evolution; and a ratio less than 1 indicates purifying (negative) selection.

### 4.7. Phylogenetic Analysis

For phylogenetic reconstruction, the six newly assembled *Lavandula* genomes were combined with 109 published chloroplast genomes from the family Lamiaceae downloaded from the National Center for Biotechnology Information (NCBI) database (Supplementary Table S2). One outgroup from the order Lamiales was included to root the tree. Whole chloroplast genome sequences were aligned using MAFFT v7.427 [45] with the --auto parameter. The resulting alignment was used as the input for maximum likelihood (ML) phylogenetic inference performed with IQ-TREE v2.2.0 [49]. The best-fit nucleotide substitution model was automatically selected using the -m MFP option. Branch support was assessed using ultrafast bootstrap approximation with 1000 replicates (-B 1000). In addition, phylogenetic trees were reconstructed using Bayesian inference in ExaBayes [50] with a DNA evolution model, utilizing a fixed random seed for reproducibility. The analysis was executed using 32 chains (-T 32) split into 4 independent runs with 4 coupled MCMCMC chains each (numruns 4, numCoupledChains 4), with state swaps performed twice per generation (numswapspergen 2) starting from non-parsimony random trees (parsimonyStart false). Runs were executed for up to 5 million generations with early stopping upon reaching convergence, which was monitored via standard ExaBayes diagnostics including ASDSF (Average Standard Deviation of Split Frequencies) and ESS (Effective Sample Size). The resulting tree was visualized using the iTOL web service [51].

To compare the phylogenetic topologies derived from Maximum Likelihood and Bayesian Inference, topological congruency analysis was performed in R using the ape [52], phangorn [53], and phytools [54] packages. Taxon labels were normalized across both trees, and topologies were pruned to a shared, identical set of taxa. Topological distance was quantified using absolute and normalized Robinson–Foulds (RF) distances calculated via the phangorn package. Visual assessment of topological concordance and clade tangles was conducted by constructing a cophylo object and plotting tanglegrams with the phytools package.

## 5. Conclusions

In this study, we successfully assembled and fully annotated six complete chloroplast genomes of *Lavandula angustifolia* (‘Hidcote’, ‘Rosea’, ‘Sineva’, ‘Stepnaya’) and *Lavandula* × *intermedia* (‘Lavandin’, ‘Lavandin Grosso’). These newly obtained chloroplast genomes significantly expand the genomic resources for the genus *Lavandula*. Fundamentally, they provide a reliable reference dataset that deepens our understanding of chloroplast genome structural evolution, maternal inheritance in interspecific hybrids, and phylogenomic relationships within the family Lamiaceae. The identified divergence hotspots and repeat profiles highlight primary candidate regions for subsequent experimental testing on larger, representative sample sets to evaluate their potential application in future molecular identification and population genetic studies.

## Figures and Tables

**Table 1:** Summary of sequencing data and assembled chloroplast genome characteristics for six *Lavandula* accessions.

Sample	Raw Reads (Million)	Cleaned Reads (Million)	GC Content of Cleaned Reads (%)	Genome Length (bp)	GC Content of Genome (%)	Genes	Mean Depth Chloroplast Genome (x)	Mean Depth IR (x)	GenBank Accession
*L. angustifolia* ‘Hidcote’	37.07	36.90	40.51	151,479	37.8	113	2440.76	2796.52	QB017885
*L. angustifolia* ‘Rosea’	32.97	32.80	41.53	151,434	37.7	113	356.68	467.93	QB017886
*L. angustifolia* ‘Sineva’	32.63	32.46	42.80	151,480	37.8	113	786.41	1129.95	QB017887
*L. angustifolia* ‘Stepnaya’	30.68	30.53	40.24	151,479	37.8	113	1503.92	1705.94	QB017888
*L.* × *intermedia* ‘Lavandin’	32.66	32.49	41.54	151,431	37.7	113	392.57	508.73	QB017889
*L.* × *intermedia* ‘Lavandin Grosso’	38.89	38.69	41.24	151,479	37.8	113	1329.25	1598.75	QB017890

## Data Availability

The chloroplast sequences of *Lavandula* varieties were deposited into the National Center for Biotechnology Information (NCBI) repository under BioProject accession number PRJNA1517218, BioSample accessions SAMN62640831 to SAMN62640836, and GenBank chloroplast genome accessions QB017885 to QB017890.

## Supplementary Materials

The following supporting information can be downloaded at https://www.mdpi.com/article/10.3390/ijms27219567/s1.
