# Supplementary material for: Chloroplast Genome Variation and Phylogenetic Relationships of Lavandula angustifolia and Lavandula × intermedia
Source: Int J Mol Sci. 2026 Oct 26;27(21):9567. doi: 10.3390/ijms27219567 (PMC13762719; doi:10.3390/ijms27219567)

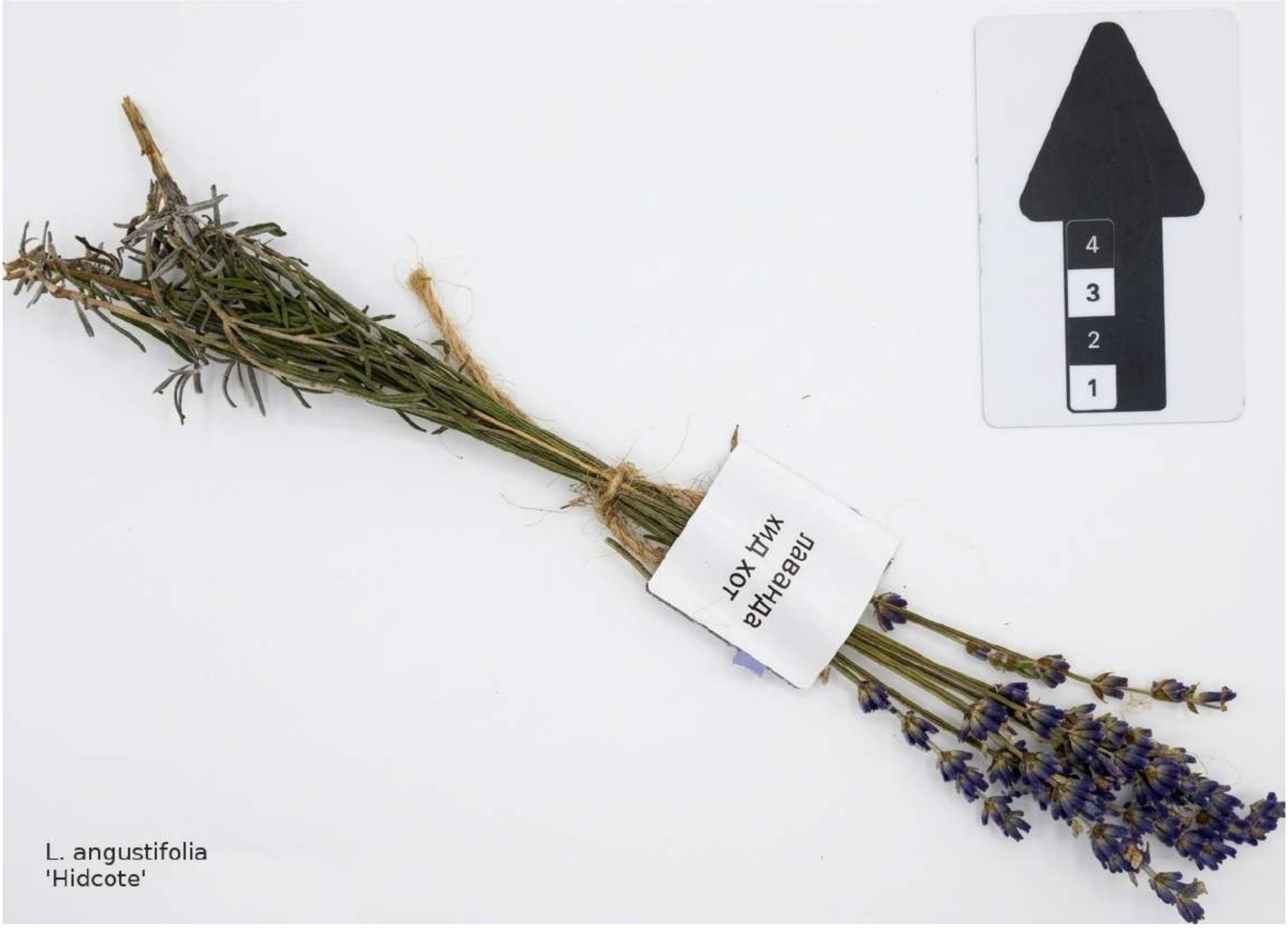

L. angustifolia
'Hidcote'

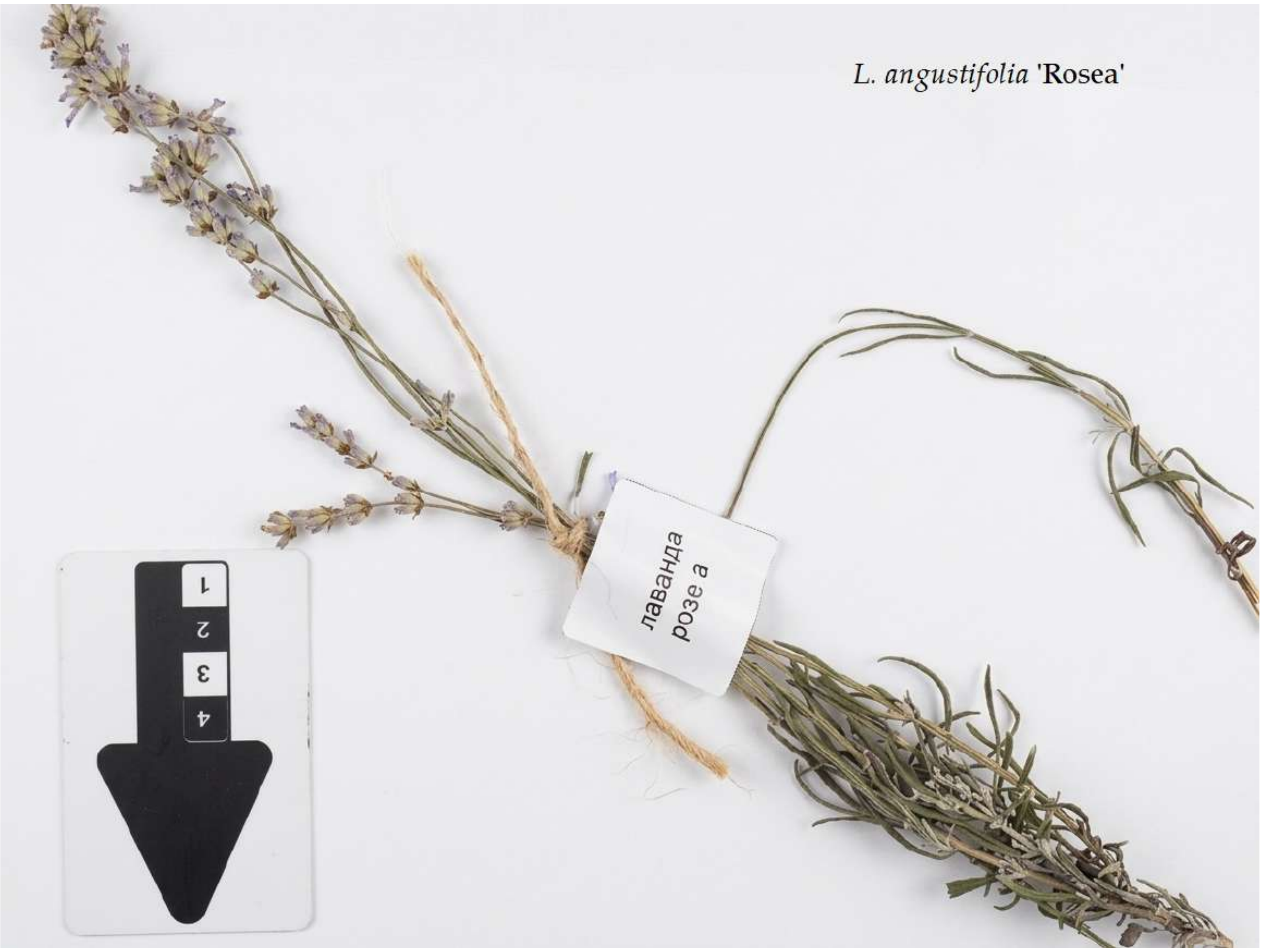

L. angustifolia 'Rosea'

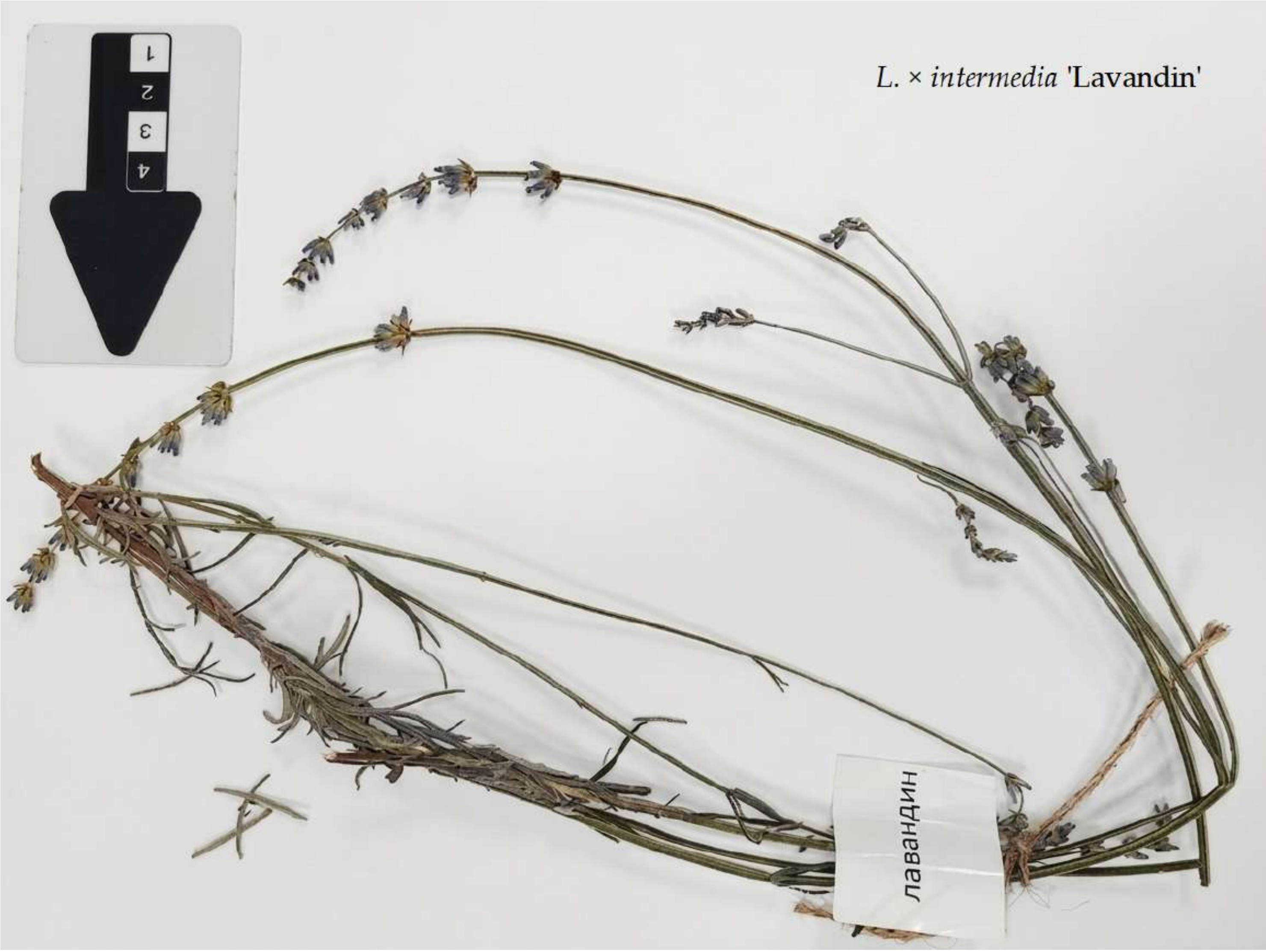
L. × *intermedia* 'Lavandin'
лавандин

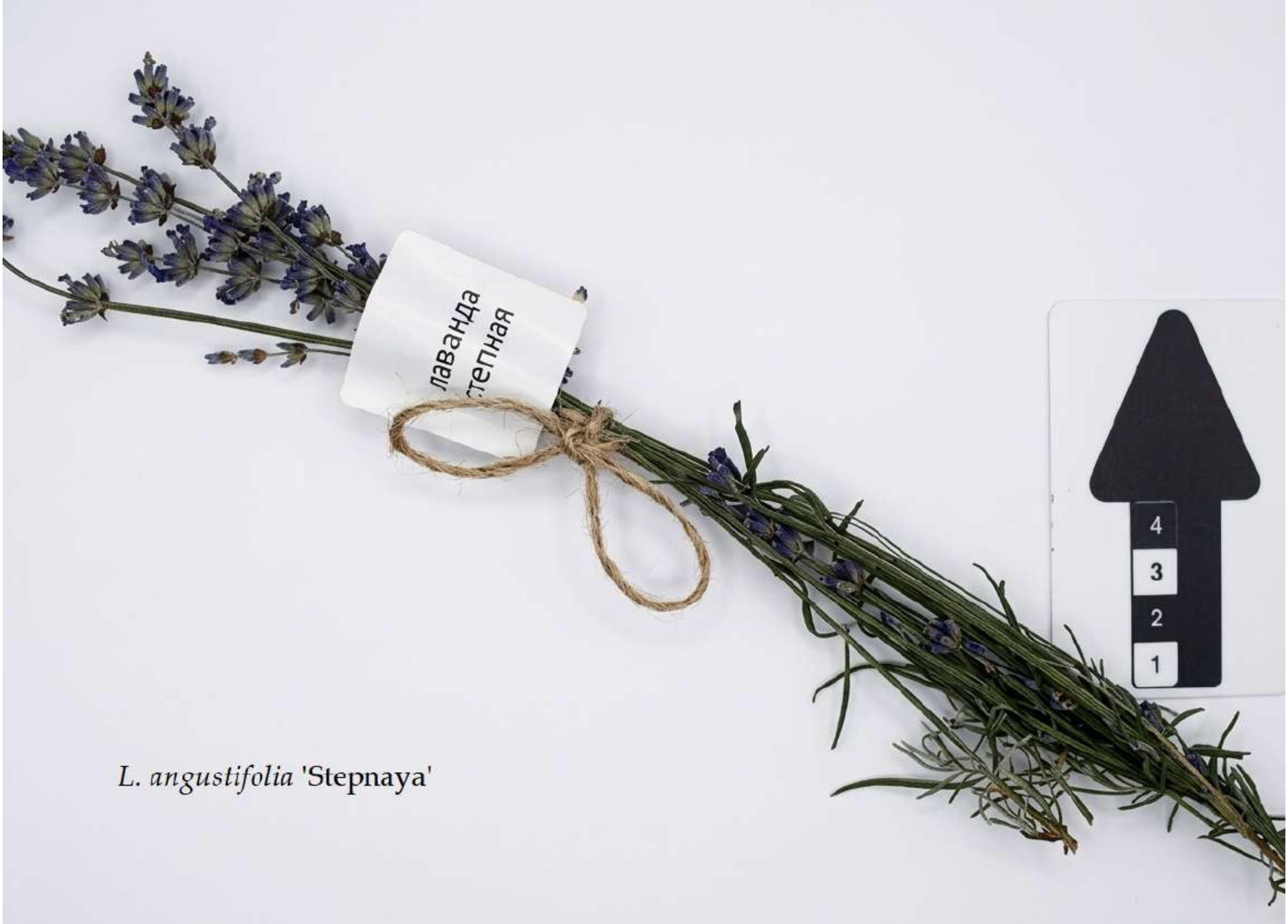

L. angustifolia 'Stepnaya'

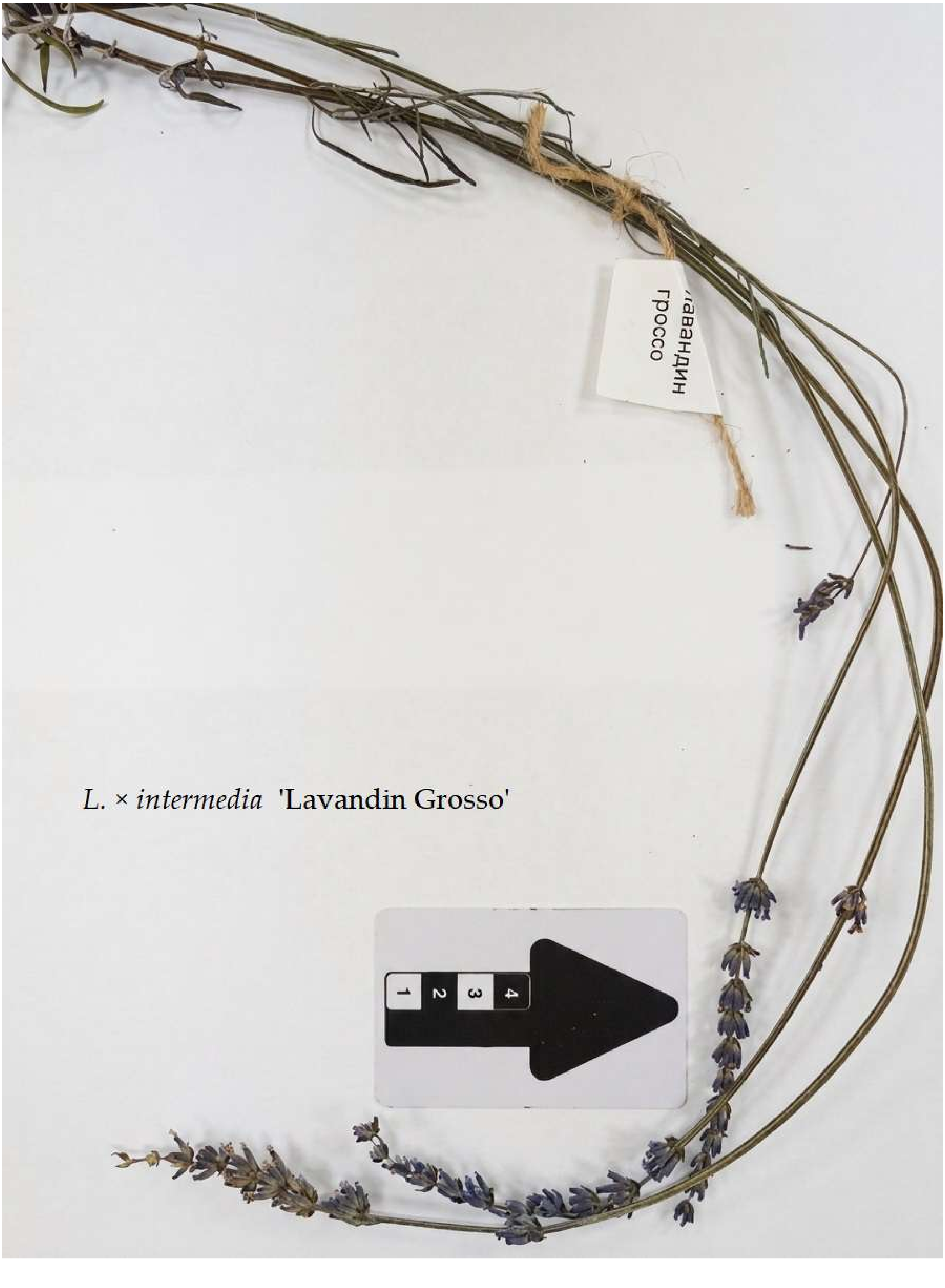

L. × *intermedia* 'Lavandin Grosso'

L. angustifolia 'Sineva'

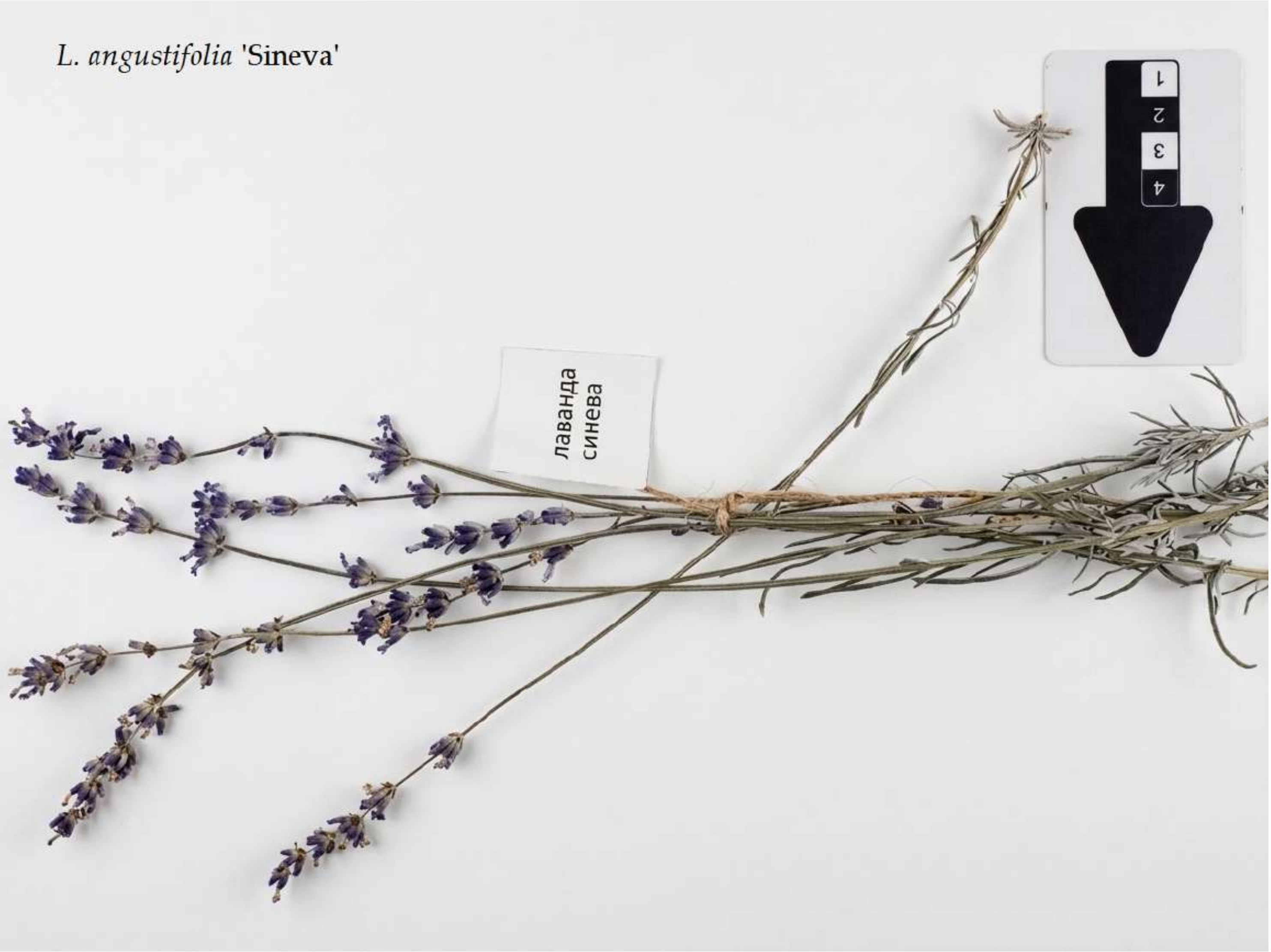

Supplement: Supplementary file 1 [file ijms-27-09567-s001.zip › FigureS8-S13.pdf]
