# Supplementary material for: Chloroplast Genome Variation and Phylogenetic Relationships of Lavandula angustifolia and Lavandula × intermedia
Source: Int J Mol Sci. 2026 Oct 26;27(21):9567. doi: 10.3390/ijms27219567 (PMC13762719; doi:10.3390/ijms27219567)
Supplement: Supplementary file 1 [file ijms-27-09567-s001.zip › Supplementary FileS1.pdf]

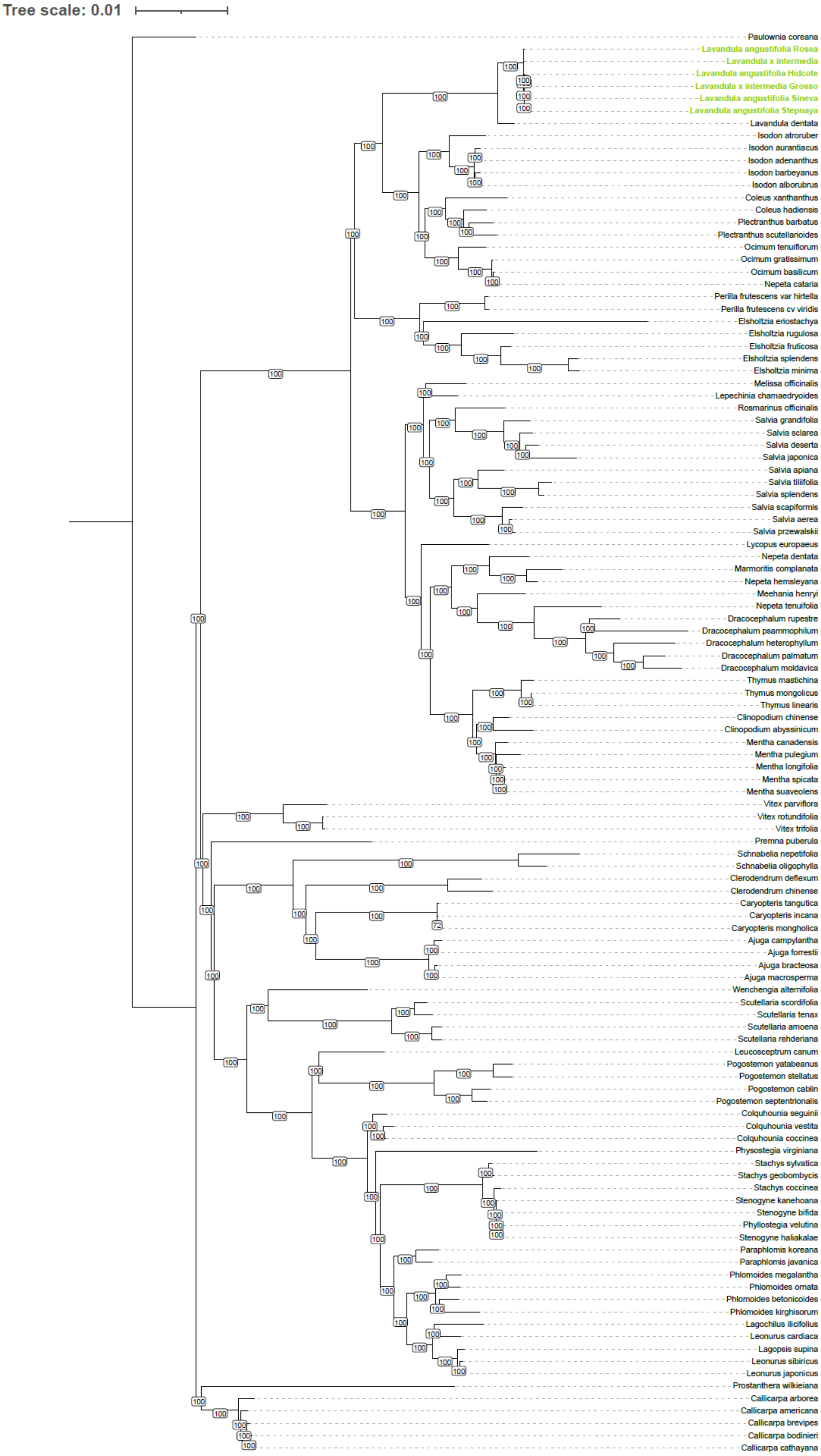

Figure S14. Bayesian Inference (BI) phylogenetic tree of the family Lamiaceae based on whole chloroplast genome sequence alignments. The tree was reconstructed using ExaBayes with *Paulownia coreana* designated as the outgroup. Bayesian posterior probability (BPP) values are displayed at the nodes.

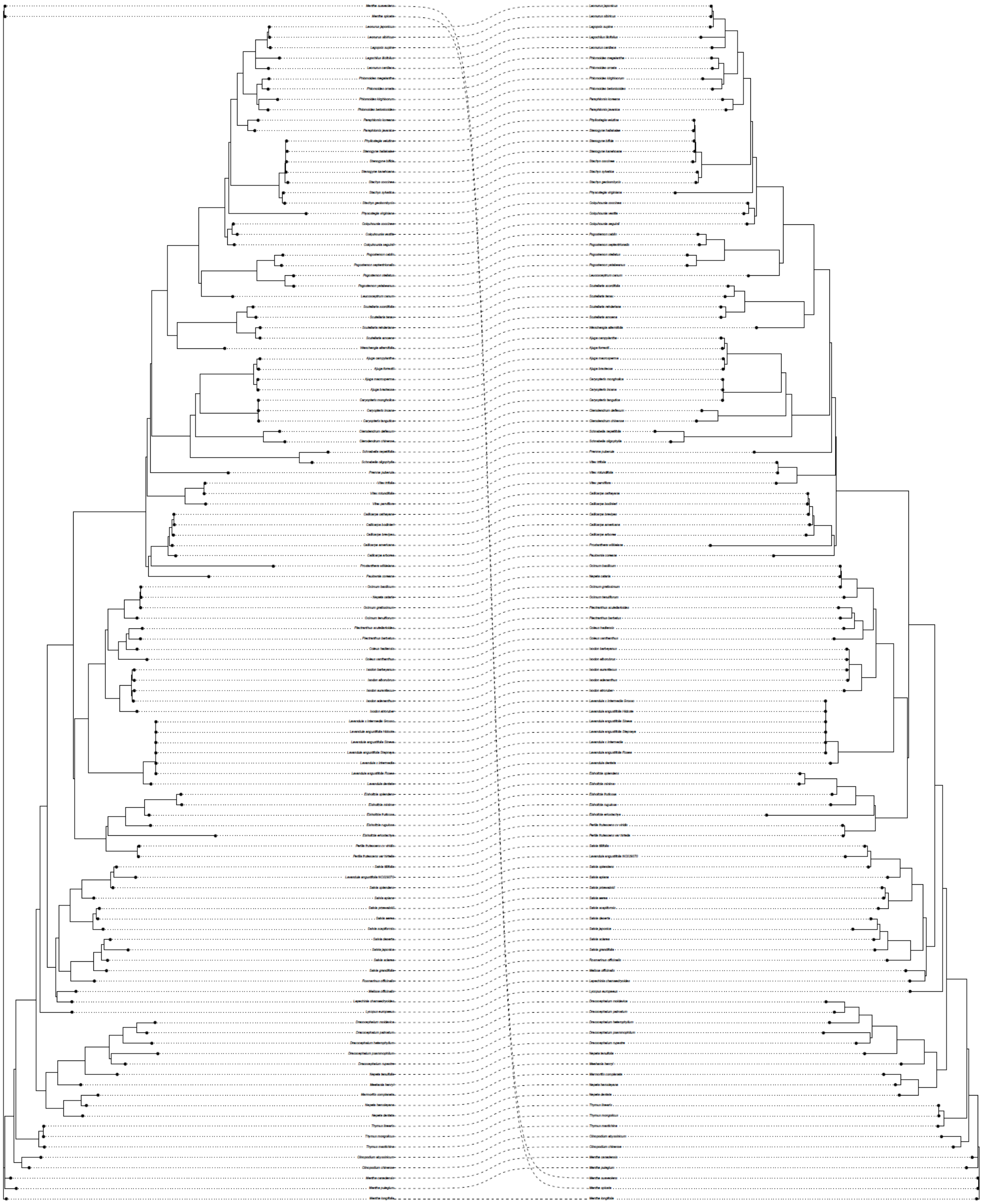

Figure S15. Tanglegram comparison evaluating topological concordance between the Maximum Likelihood (ML; left) and Bayesian Inference (BI; right) phylogenetic trees of Lamiaceae. Both trees were reconstructed from the shared 115-taxon whole chloroplast genome alignment, rooted with *Paulownia coreana*s. Auxiliary connecting lines link corresponding taxa across the two phylogenies. The comparison reveals absolute topological congruence (normalized Robinson–Foulds distance, RF = 0) with no conflicting clade tangles between the two optimality criteria
